# Pulsating hemorrhagic varicose veins caused by tricuspid valve regurgitation: report of a case treated by laser ablation and foam sclerotherapy

**DOI:** 10.1186/s40792-021-01289-2

**Published:** 2021-09-03

**Authors:** Atsushi Guntani, Sho Yamashita, Shinsuke Mii

**Affiliations:** Department of Vascular Surgery, Saiseikai Yahata General Hospital, 5-9-27 Haruno-machi, Yahatahigashi-ku, Kitakyushu, 805-8527 Japan

**Keywords:** Endovenous laser ablation, Varicose vein, Tricuspid valve regurgitation

## Abstract

**Background:**

Varicose veins are one of the most common disease; however, secondary varicose veins caused by tricuspid valve regurgitation (TVR) are rare.

**Case presentation:**

A patient who developed pulsating bleeding from superficial varicose veins due to TVR was successfully treated by endovenous laser ablation (EVLA) of the great saphenous vein (GSV) and repeated foam sclerotherapy of varicose veins. There were no complications, such as rebleeding or recanalization of the GSV during the 1-year follow-up period.

**Conclusions:**

We herein report a rare case of pulsatile hemorrhagic varicose veins caused by TVR that was successfully managed by combined treatment of EVLA and foam sclerotherapy. When pulsatile varicose veins are found, the presence of TVR should be suspected.

## Background

Most varicose veins are caused by primary valvular insufficiency. It is reported that only about 5–20% of varicose veins occur secondary to conditions, such as deep vein thrombosis, pelvic tumor or arteriovenous fistula [1, 2]. In particular, secondary varicose veins caused by tricuspid valve regurgitation (TVR) are extremely rare [3–5]. We successfully treated a patient with pulsatile hemorrhagic varicose veins caused by TVR using endovenous laser ablation (EVLA) of the great saphenous vein (GSV) and repeated foam sclerotherapy of varicose veins.

## Case presentation

**Fig. 1:**
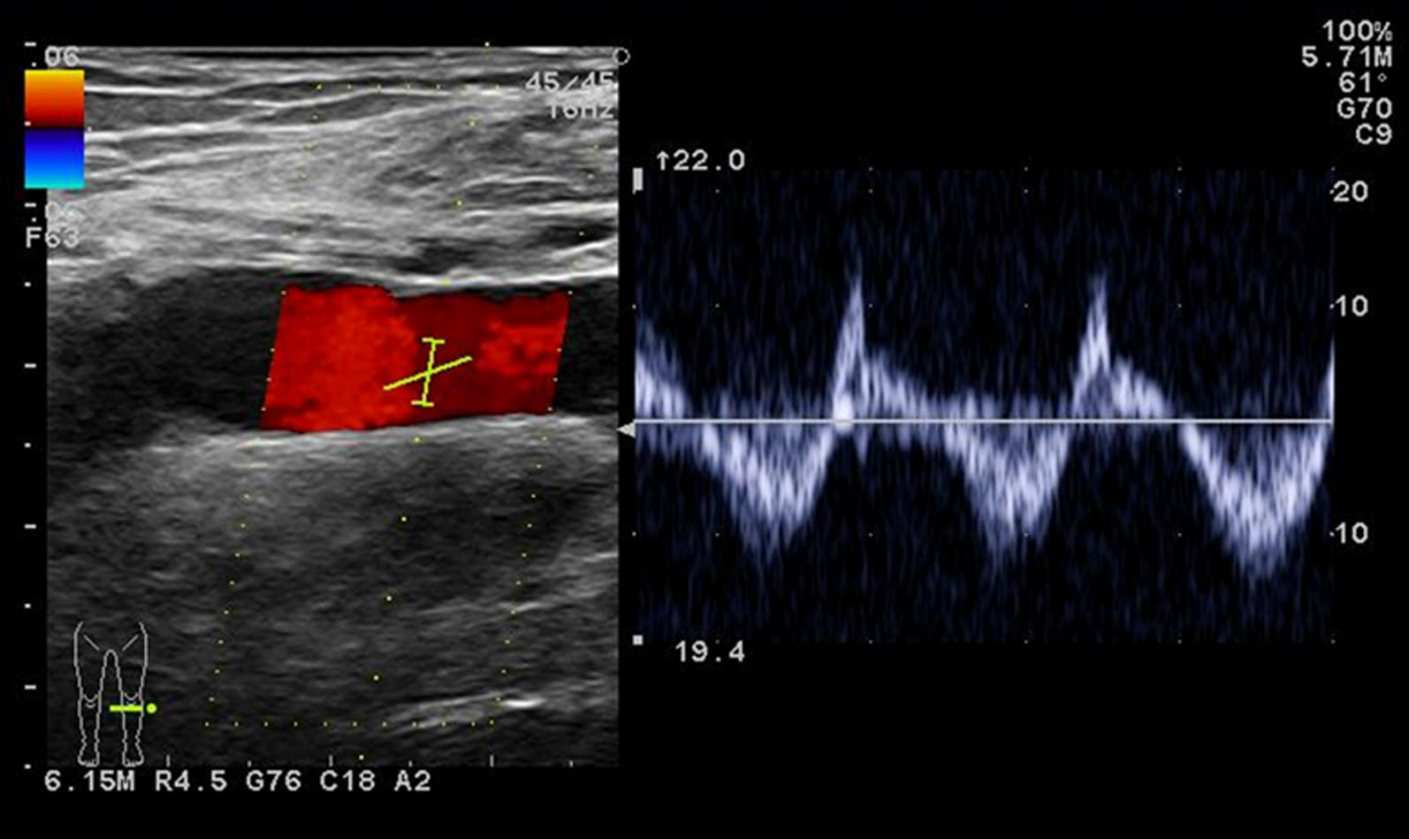
Ultrasonography showed pulsatile regurgitation in the left GSV

A 69-year-old male patient was admitted for pulsating bleeding from superficial varicose veins of the left dorsal foot. After compression hemostasis, ultrasonography revealed pulsatile regurgitation in the left GSV (Fig. 1). He had a history of ligation of two incompetent perforating veins in the left thigh, and high ligation and stripping of the right GSV. In a previous clinical chart, pulsatile palpation of the right GSV with significant postoperative hematoma was described in the surgical record; however, no further examination was performed at that time.

**Fig. 2:**
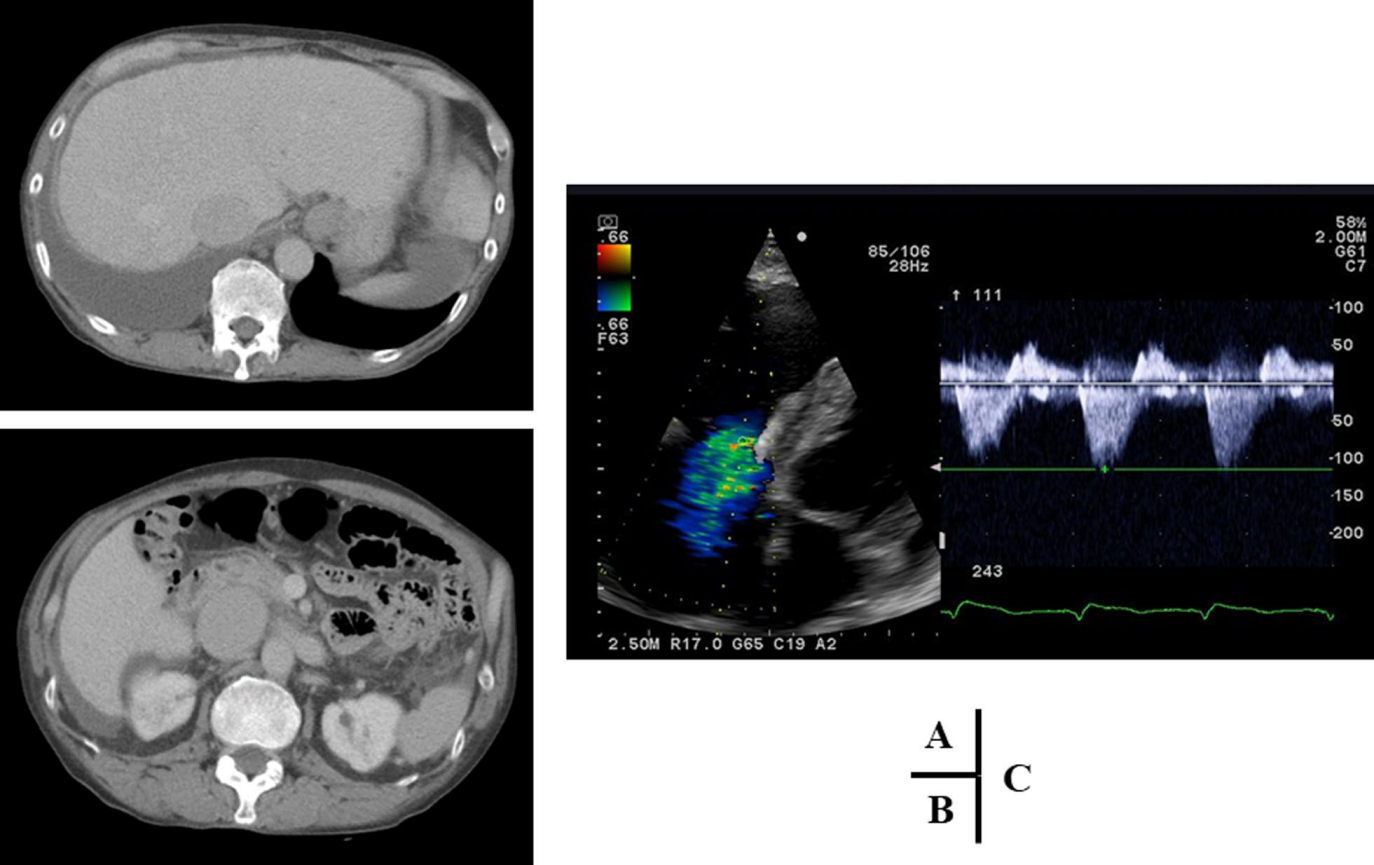
Computed tomography revealed the presence of congestive liver and inferior vena cava dilatation (**A**, **B**). Echocardiography showed prominent enlargement of the right atrium and right ventricle, and severe TVR (**C**)

Dilated superficial varicose veins and pigmentation were observed from the left lower leg to the dorsal foot, equivalent to C_4a_, as assessed using the clinical, etiological, anatomical and pathological (CEAP) classification. Ultrasonography revealed regurgitation in the left deep veins, from the common femoral to the popliteal, and perforators in the left lower leg, in addition to the left GSV. We clinically suspected arteriovenous fistula and the patient was admitted for a detailed hemodynamic examination. Computed tomography revealed the presence of congestive liver and inferior vena cava dilatation; however, no arteriovenous fistula was observed (Fig. 2A, B). Subsequent echocardiography showed prominent enlargement of the right atrium and right ventricle, and severe TVR (Fig. 2C). Thus, the pulsatile venous blood flow was considered to be derived from TVR.

**Fig. 3:**
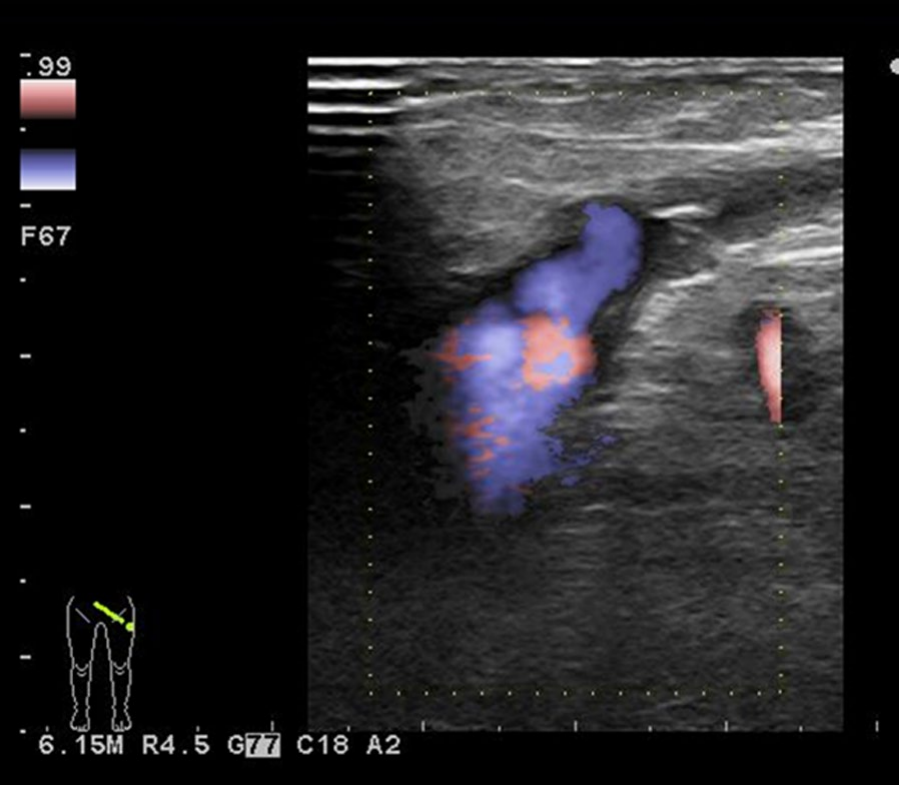
Follow-up duplex sonography revealed that the success of EVLA of the left GSV and the patency of the deep vein system

EVLA was performed on the left GSV of the thigh using a 1470 nm diode laser with a radial 2-ring fiber (ELVeS Radial 2ring™ fiber, CeramOptec GmbH, Germany) under general anesthesia with tumescent local anesthesia (430 mL saline + 50 mL lidocaine 1% + 20 mL sodium hydrotricarbonate 7%). Subsequently, three incompetent perforating veins in the left lower leg were identified under ultrasound guidance, and then ligated and dissected. The puncture site of the introducer sheath and the incision in the lower leg bled easily; however, hemostasis could be obtained by manual compression. After the operation, the patient rested on the bed with the left lower limb compressed by an elastic bandage. Duplex sonography confirmed the success of EVLA of the left GSV and the patency of the deep vein system (Fig. 3). Two days after EVLA, foam sclerotherapy using 1% polidocanol (dilution ratio for sclerosant to air: 1:3) was performed for the superficial varicose veins of the left foot, including the bleb, where bleeding was encountered at the time of admission.

The patient used a compression stocking and was followed regularly as an outpatient. It was necessary to perform foamed sclerotherapy three times for other superficial varicose veins of the left lower limb; however, the examination using duplex ultrasonography had not detected recanalization of the occluded left GSV and bleeding from the varicose veins was not observed during the 1-year follow-up period.

## Discussion

Tricuspid insufficiency results in elevated central venous pressure, and various clinical signs, such as enlarged liver, peripheral edema, and ascites are observed. According to past case reports, in cases of tricuspid insufficiency, pulsatile veins have been observed in the neck, forearm, and forehead [6, 7]. In our case, computed tomography revealed congestive liver, pleural effusion, and ascites; however, there were no obvious subjective symptoms other than pulsating bleeding from the superficial varicose veins of the left foot.

Our patient had a history of high ligation and stripping of the right GSV. The right GSV was pulsating according to the surgical record. Significant hematoma occurred as a complication after the operation. It seems that TVR was already present; however, no further examination was performed. Right ventricular cardiomyopathy caused by arrhythmia was the main pathology of the disease and the main cause of right ventricular enlargement and decreased right ventricular contractility, which led to TVR. Therefore, it was highly possible that even if tricuspid valve replacement surgery had been performed, it would not have contributed to the reduction in the venous pressure. Drug therapy with diuretics (torsemide, 8 mg, daily) and cardiac stimulants (pimobendane, 5 mg, daily) was introduced after the bleeding event involving the varicose veins.

In previous studies, some cases of pulsatile varicose veins secondary to TVR were treated by approaches, such as EVLA alone and saphenofemoral ligation with foam sclerotherapy; however, other cases were treated conservatively by compression therapy with elastic stockings, in consideration of the bleeding due to venous hypertension and cardiac risk during the operation [1, 8–11]. Our case has a history of both stripping and EVLA of the GSV; however, EVLA seems to be associated with a lower risk of bleeding, considering the complication of hematoma after stripping of the GSV. In addition, since the course of this case was favorable during the 1-year follow-up period, it was considered that EVLA showed satisfactory venous sealing efficiency, even in the presence of high venous pressure.

Deep vein regurgitation was also observed in our case, so even after EVLA of the GSV, regurgitation through the incompetent perforating vein and rebleeding from varicose veins may occur. Therefore, it was considered that ligation of the incompetent perforating veins and foam sclerotherapy for the superficial varicose veins were effective. Since there is not enough evidence concerning the long-term outcomes of EVLA for varicose veins caused by TVR, further follow-up will be necessary.

## Conclusion

We herein report a rare case of pulsatile hemorrhagic varicose veins caused by TVR that was successfully treated by EVLA and foam sclerotherapy. When pulsatile varicose veins are found, the presence of TVR should be suspected.

## Data Availability

All data sets supporting the conclusions of this article are included in this published article.

## Abbreviations

TVR: Tricuspid valve regurgitation
GSV: Great saphenous vein
EVLA: Endovenous laser ablation
CEAP: Clinical, etiological, anatomical and pathological
